# Interplay Between Sperm Function and Seminal Plasma Biochemical Profile in a Group of Normozoospermic Subjects

**DOI:** 10.3390/biom16081125

**Published:** 2026-08-01

**Authors:** Caterina Marcucci, Giulia Collodel, Elena Leoni, Laura Liguori, Cinzia Signorini, Fabiola Nerucci, Paola Calzoni, Marcello Fiorini, Elena Moretti

**Affiliations:** 1 Department of Molecular and Developmental Medicine, University of Siena, 53100 Siena, Italy; caterin.marcucci@student.unisi.it (C.M.); elena.leoni@student.unisi.it (E.L.); laura.liguori@student.unisi.it (L.L.); cinzia.signorini@unisi.it (C.S.); elena.moretti@unisi.it (E.M.); 2 Division of Clinical Pathology, University Teaching Hospital of Siena, 53100 Siena, Italy; fabiola.nerucci@gmail.com (F.N.); p.calzoni@ao-siena.toscana.it (P.C.); marcello.fiorini@ao-siena.toscana.it (M.F.)

**Keywords:** homocysteine, normozoospermia, oxidative stress, seminal plasma biochemical components, sperm quality, Trolox Equivalent Antioxidant Capacity (TEAC)

## Abstract

Seminal plasma regulates sperm function; oxidative stress (OS) and inflammation can impair semen biochemical homeostasis. This study investigated seminal biochemical/enzymatic compounds and Trolox Equivalent Antioxidant Capacity (TEAC) in seminal plasma and spermatozoa from normozoospermic men (>5th percentile) assessing their relationship with sperm quality. The strongest significant (*p* < 0.001 ***) negative correlations (r) were found for normal sperm morphology and albumin (ALB, r = −0.515 ***), cholesterol (CHO, r = −0.386 ***), lactate dehydrogenase (LDH, r = −0.514 ***), sperm TEAC (r = −0.438 ***) and homocysteine (Hcy; r = −0.381 ***); sperm progressive motility with ALB (r = −0.495 ***), CHO (r = −0.349 ***), LDH (r = −0.355 ***) and sperm TEAC (r = −0.295 ***); sperm vitality with creatine kinase (CK, r = −0.486 ***) and LDH (r = −0.380 ***). Total prostate-specific antigen (TPSA) was positively correlated with sperm quality. After stratification, subjects with low-normal sperm parameters showed significantly higher levels of ALB, CHO, CK, LDH, interleukin 6 (IL-6), Hcy and sperm TEAC vs. high-normal parameter subjects. ALB, Hcy and LDH were increased in individuals with progressive motility, normal morphology and vitality < 25th percentile indicating their involvement in OS, inflammation and accessory gland activity. These findings suggest that World Health Organization lower reference limits are not a strict cutoff between normal and abnormal semen quality but rather define a baseline of biochemical efficiency and sperm function, highlighting the synergy between spermatozoa and seminal plasma.

## 1. Introduction

Seminal plasma is enriched in molecules critical for sperm function, including basic amines, carbohydrates, lipids, vitamins, minerals, proteins, cytokines and nucleic acids [1,2,3]. This molecular complexity underlies several essential functions of seminal plasma in sperm protection, maturation, capacitation and interactions with the female reproductive tract, reflecting the functional interdependence between the cellular and fluid components of semen [4]. Consequently, seminal plasma has garnered increasing attention as a potential source of biomarkers for the diagnosis and monitoring of male reproductive disorders [3,5].

Recent research has been directed toward the detailed characterization of the enzymatic, biochemical composition of semen [6,7,8,9,10,11], metabolome, exploring their association with sperm quality [12,13,14].

Several seminal molecules, particularly relevant to male reproductive physiology, encompassing proteins and metabolites involved in cellular metabolism, oxidative stress (OS), and inflammation have been considered in this paper, in particular albumin (ALB), carcinoembryonic antigen (CEA), cholesterol (CHO), creatine kinase (CK), interleukin-6 (IL-6), lactate dehydrogenase (LDH), total prostate-specific antigen (TPSA), uric acid (UA), homocysteine (Hcy) and Trolox Equivalent Antioxidant Capacity (TEAC) measured in seminal plasma and spermatozoa. ALB facilitates sperm capacitation by promoting CHO efflux from the sperm plasma membrane, enabling membrane reorganization and activation of signaling pathways required for hyperactivation and fertilization [15]. CHO represents a major structural component of the sperm plasma membrane, regulating the activity of ion channels involved in sperm capacitation [16]. Recently, alterations in CHO metabolism have also been associated with OS-induced damage and impaired fertilizing capacity [17]. The CK participates in the phosphocreatine system, sustaining sperm motility and flagellar activity [18], while LDH, particularly the sperm-specific LDH-C isoform, plays a central role for normal motility and fertilization capacity [19,20].

Inflammatory mediators are also known to influence the seminal microenvironment and sperm physiology. IL-6 is a pleiotropic cytokine involved in immune regulation and is consistently detected in human semen, even in the absence of overt infection [21]. Elevated concentrations of IL-6 in seminal plasma have been associated with increased levels of reactive oxygen species (ROS) and lipid peroxidation in spermatozoa, leading to impaired sperm quality [22]. Another molecule of particular interest is Hcy, a sulphur-containing amino acid generated during methionine metabolism. Alterations in Hcy metabolism may negatively influence semen quality [23]. Additional molecules of prostatic origin, such as CEA, have also been detected in seminal plasma. Although the physiological role in male reproduction remains poorly understood, its inclusion in the present panel was motivated by the potential value as an indicator of accessory gland contribution to the seminal microenvironment [24,25].

Moreover, TPSA, a serine protease produced by the prostate, plays a crucial role in the liquefaction of seminal plasma after ejaculation. This enzymatic process is necessary for the degradation of seminal coagulum proteins and for the release of spermatozoa, thereby facilitating sperm motility and progression within the female reproductive tract [26,27].

In seminal plasma the antioxidant capacity also plays a critical role in maintaining sperm integrity. UA, a major non-enzymatic antioxidant in human semen, contributes to preserving sperm motility, vitality and morphology, supporting overall sperm function [28,29]. Despite its recognized importance, seminal UA concentrations remain inconsistently reported, reflecting inter-individual variability and the influence of both physiological and pathological factors [30,31,32]. On the other hand, total antioxidant capacity (TAC) reflects the cumulative activity of enzymatic and non-enzymatic antioxidants present in both seminal plasma and sperm cells, which act synergistically to neutralize ROS and protect spermatozoa from oxidative damage [33]. TAC assays are frequently used to identify the antioxidant capacity also in male infertility [34]. Here, the TEAC assay was applied as a measure of antioxidant capacity expressed in Trolox equivalents [35].

Although several seminal components have been individually associated with sperm function, the integrated contribution of biochemical, enzymatic, inflammatory and OS-related markers to sperm quality remains insufficiently characterized, particularly in normozoospermic men. A comprehensive assessment of these parameters may provide new insights into the biological variability underlying apparently normal semen profiles.

Based on these observations, the present study aimed to investigate in a cohort of normozoospermic individuals (>5th percentile; World Health Organization (WHO) [36]) the seminal concentrations of biochemical and enzymatic components and TEAC levels in seminal plasma and spermatozoa. This analytical approach can indicate whether these markers may vary also in relation to sperm parameters, always within the condition of normozoospermia.

## 2. Materials and Methods

### 2.1. Participants and Groups

**Figure 1:**
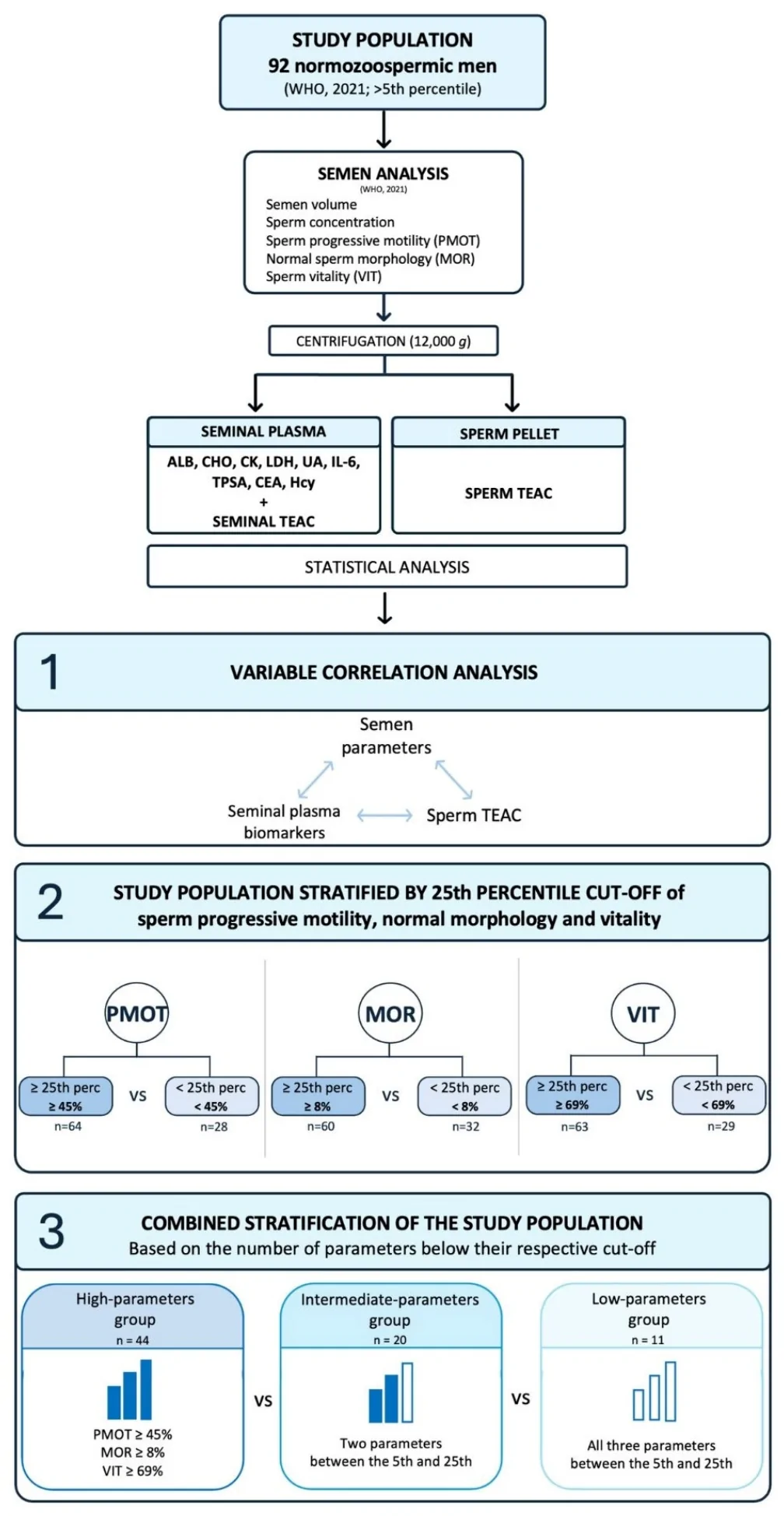
Study design and stratification of the study population. A total of 92 normozoospermic men were enrolled according to the WHO reference criteria [36] and additional inclusion criteria. Semen samples underwent standard semen analysis; subsequently, seminal plasma was used to measure albumin (ALB), carcinoembryonic antigen (CEA), cholesterol (CHO), creatine kinase (CK), interleukin-6 (IL-6), lactate dehydrogenase (LDH), total prostate-specific antigen (TPSA), uric acid (UA), homocysteine (Hcy) and Trolox Equivalent Antioxidant capacity (TEAC), whereas the sperm pellet was used for sperm TEAC assessment. Following correlation analyses in the whole study population, subjects were first stratified according to the 25th percentile of sperm progressive motility, normal sperm morphology and sperm vitality, generating the PMOT < 25/PMOT ≥ 25, MOR < 25/MOR ≥ 25 and VIT < 25/VIT ≥ 25 comparison groups, respectively. Subsequently, the same cohort was classified into high- (H), intermediate- (I), and low-parameters (L) groups according to the combined distribution of sperm progressive motility, morphology and vitality values between the 5th and 25th percentiles or above the 25th percentile.

The study group consisted of 92 normozoospermic men (aged 27–41 years) attending the Department of Molecular and Developmental Medicine for semen analysis. All individuals exhibited semen parameters above the 5th percentile according to WHO guidelines [36] and provided the required anamnestic information. Additional inclusion criteria were: a normal karyotype, BMI < 25 kg/m^2^, and normal serum hormone levels. The individuals had no urogenital infections, leukocytospermia, and did not smoke more than 10 cigarettes per day. They did not report chronic diseases, chemotherapy, radiotherapy, intake of oral antioxidant supplementation, or a history of drug or alcohol use. To investigate whether, within normozoospermia (above the 5th percentile [36]), specific seminal parameters correlate with the level of specific biochemical molecules measured in seminal plasma, values of sperm progressive motility, normal sperm morphology and sperm vitality corresponding to the 25th percentile [36] were used to stratify the selected normozoospermic individuals as follows (Figure 1):

- The group of individuals with sperm progressive motility below the 25th percentile (PMOT < 25 group) included 28 normozoospermic men with progressive motility < 45%, whereas the group with sperm progressive motility ≥ 25th percentile (PMOT ≥ 25 group) included 64 normozoospermic men with progressive motility ≥ 45% [36];
- The group of individuals with normal sperm morphology below the 25th percentile (MOR < 25 group) included 32 normozoospermic men with normal morphology < 8%, whereas the group with normal sperm morphology ≥ 25th percentile (MOR ≥ 25 group) included 60 normozoospermic men with normal morphology ≥ 8% [36];
- The group of individuals with sperm vitality below the 25th percentile (VIT < 25 group) included 29 normozoospermic men with vitality < 69%, whereas the group with sperm vitality ≥ 25th percentile (VIT ≥ 25 group) included 63 normozoospermic men with vitality ≥ 69% [36].

Subsequently, the same cohort of normozoospermic individuals was stratified into three groups:

- High-parameter group (H, n = 44), in whom all three sperm parameters were greater than or equal to the 25th percentile (respectively sperm progressive motility ≥ 45%, normal sperm morphology ≥ 8%, and sperm vitality ≥ 69%);
- Intermediate-parameter group (I, n = 20), defined by having two out of the three evaluated parameters are comprised between the 5th and 25th percentile (respectively sperm progressive motility 30–45%, normal sperm morphology 4–8%, and sperm vitality 54–69%);
- Low-parameter group (L, n = 11), in whom all three parameters fell between the 5th and 25th percentiles (respectively 30–44%, 4–7%, and 54–68%).

Subjects with only one of the three selected sperm parameters at or above the 25th percentile and the remaining two parameters between the 5th and 25th percentiles did not meet the predefined classification criteria for the H, I, or L groups and were not included in the corresponding group-based analyses.

The 25th percentile was chosen as a data-driven, exploratory threshold to identify the lowest-performing subgroup and assess whether specific biochemical markers are preferentially associated with reduced sperm parameters. It also provided an intermediate reference point while ensuring an adequate distribution of subjects among the three groups for comparative analyses.

All patients agreed to have their semen analyzed for semen components and for assessing OS. The study was approved by the Ethics Committee of Azienda Ospedaliera Universitaria Senese (ID: CEAVSE 25612, 20 November 2023).

### 2.2. Semen Analysis

Semen analysis was performed according to WHO guidelines [36]. Briefly, semen samples were collected by masturbation after 3–5 days of sexual abstinence and analyzed after liquefaction for 30 min at 37 °C. Conventional semen parameters were determined: volume, pH, sperm concentration, motility, and vitality. The sperm morphology was assessed by the stain-coated test simplet slides (Origio, Italy). Sperm vitality was assessed using Eosin Y staining by evaluating at least 300 spermatozoa per sample and distinguishing viable (unstained) from non-viable (red-stained) cells.

After semen analysis, all samples were centrifuged at 12,000× *g* to separate spermatozoa from the seminal plasma, according to Feng and colleagues [37]. The recovered seminal plasma was examined under a light microscope to confirm the absence of sperm cells. The sperm pellet was washed three times with phosphate-buffered saline (PBS), and aliquots containing approximately 1 × 10^6^ spermatozoa were prepared for other analyses. Both seminal plasma and sperm aliquots were stored at −80 °C until use.

The samples were stored for 3–5 months before the analysis. Upon thawing, they were immediately processed and were not subjected to any further freeze–thaw cycles.

### 2.3. Determination of Biochemical Components in Seminal Plasma

The stored seminal plasma specimens were thawed at room temperature and then tested using a COBAS 8000 modular analyzer (Roche Diagnostics GmbH, Mannheim, Germany) by means of two analytical modules: C702, the high-throughput clinical chemistry module, and E602, the immunoassay module. The following parameters were measured in seminal plasma: ALB, g/dL, CEA, ng/mL, CHO, mg/dL, CK, U/L, IL-6, pg/mL, LDH, U/L, TPSA, ng/mL, UA, mg/dL. For the analytes measured in module C702 (ALB, LDH, CK, CHO, UA), COBAS 8000 calibration was done with the human lyophilized serum Calibrator C.f.a.s. (Roche Diagnostics GmbH, Mannheim, Germany). The C.f.a.s. (calibrator for automated systems) is a universal calibrator for adjusting most photometric methods. Human lyophilized serum PreciControl ClinChem level 1 was used as normal control and PreciControl ClinChem level 2 was used as pathologic control (Roche Diagnostics GmbH, Mannheim, Germany). For the analytes measured in module E602 (CEA, IL-6, TPSA), specific calibrators for each analyte (Roche Diagnostics GmbH, Mannheim, Germany) were used. PreciControl Tumor Marker levels 1 and 2 (Roche Diagnostics GmbH, Mannheim, Germany), and Preci control Multimarker levels 1 and 2 (Roche Diagnostics GmbH, Mannheim, Germany) were used as normal and pathologic controls respectively.

Then, semen samples were centrifuged at 2600× *g* for 15 min and the resulting supernatant was used for Hcy quantification by nephelometric assay on the Atellica NEPH 630 System (Siemens Healthineers, Milano Italy), employing the N Latex HCY reagent (Siemens Healthineers, Milano Italy). Concentrations were calculated using a calibration curve generated with the N Protein Standard SL (Siemens Healthineers, Milano, Italy), and results were expressed in micromoles per liter (µmol/L). Each analytical session was validated through internal quality control using the N/T Protein Control SL/M (Siemens Healthineers, Milano, Italy).

### 2.4. Trolox Equivalent Antioxidant Capacity Determination in Seminal Plasma and Spermatozoa

The total antioxidant capacity of seminal plasma and spermatozoa was quantified using an antioxidant assay kit (Sigma-Aldrich, St. Louis, MO, USA). After thawing, spermatozoa were suspended in 0.5 mL of cold 1× Assay Buffer and sonicated to prepare cell lysates. Afterward, a centrifugation at 12,000× *g* for 15 min at 4 °C was carried out and the supernatant was collected. Seminal plasma was thawed at room temperature and analyzed. The applied antioxidant assay measures the ability of sample antioxidants to inhibit the formation of the radical cation of 2,2′-azino-bis (3-ethylbenzothiazoline-6-sulfonic acid) (ABTS•+) generated by the reaction of metmyoglobin with hydrogen peroxide. In the presence of antioxidants, the production of ABTS•+ is suppressed in a concentration-dependent manner, resulting in a reduction in the green color intensity, which was measured spectrophotometrically at 405 nm. Trolox, a water-soluble vitamin E analog, was used to generate a standard curve, and results, reported as mM concentrations, were expressed as TEAC in both seminal plasma (seminal TEAC) and spermatozoa (sperm TEAC).

### 2.5. Statistical Analysis

The D’Agostino–Pearson test was used to assess data distribution. Medians and interquartile ranges (IQR: 25th–75th percentile) of the considered variables were used to represent the data. Spearman’s rank correlation coefficient was used to assess the correlations among the variables under investigation in the overall study population. Comparisons between the PMOT < 25 and PMOT ≥ 25 groups, MOR < 25 and MOR ≥ 25 groups and VIT < 25 and VIT ≥ 25 groups were performed using the Mann–Whitney U test. Comparisons among the high-, intermediate- and low (H, I, L)-parameter groups were performed using the Kruskal–Wallis test followed by Dunn’s post hoc test for multiple comparisons when a statistically significant difference was found among groups. A *p* value < 0.05 was considered statistically significant.

## 3. Results

Seminal levels of ALB, CEA, CHO, CK, IL-6, LDH, TPSA, UA and Hcy were quantified in semen samples of 92 individuals with normal semen parameters (>5th percentile, [36]). TEAC was assessed in both seminal plasma and spermatozoa (seminal TEAC and sperm TEAC).

In Table 1, the medians and interquartile range (IQR, 25th–75th percentile) for each variable are shown.

The correlations between the variables under investigation in the overall study population are reported in Table 2.

The biochemical profile of the seminal plasma revealed a complex network of interactions. Among seminal biomarkers, ALB showed positive significant correlations with CHO (r = 0.377, *p* < 0.001), LDH (r = 0.577, *p* < 0.001) and Hcy (r = 0.426, *p* < 0.001), and a negative correlation with TPSA (r = −0.425, *p* < 0.001). CEA showed positive correlations with TPSA (r = 0.245, *p* < 0.05), UA (r = 0.233, *p* < 0.05) and Hcy (r = 0.209, *p* < 0.05). CHO was positively correlated with LDH (r = 0.582, *p* < 0.001) and sperm TEAC (r = 0.223, *p* < 0.05) and negatively with TPSA (r = −0.530, *p* < 0.001) and UA (r = −0.346, *p* < 0.001). CK showed positive correlations with IL-6 (r = 0.305, *p* < 0.01) and sperm TEAC (r = 0.248, *p* < 0.05) and negative correlation with TPSA (r = −0.311, *p* < 0.01). IL-6 was positively correlated with LDH (r = 0.228, *p* < 0.05) and UA (r = 0.221, *p* < 0.05) and negatively with seminal TEAC (r = −0.213, *p* < 0.05). LDH was negatively correlated with TPSA (r = −0.457, *p* < 0.001) and positively with Hcy (r = 0.254, *p* < 0.05). UA showed positive correlations with TPSA (r = 0.338, *p* < 0.001), Hcy (r = 0.320, *p* < 0.01) and seminal TEAC (r = 0.221, *p* < 0.05).

Moreover, several biochemical markers exhibited significant relationships with sperm parameters.

Normal sperm morphology showed significant negative correlations with ALB (r = −0.515, *p* < 0.001), CHO (r = −0.386, *p* < 0.001), LDH (r = −0.514, *p* < 0.001), Hcy (r = −0.381, *p* < 0.001), sperm TEAC (r = −0.438, *p* < 0.001), CK (r = −0.294, *p* < 0.01) and CEA (r = −0.207, *p* < 0.05) and a positive correlation with TPSA (r = 0.258, *p* < 0.05).

Progressive sperm motility had significant negative correlations with ALB (r = −0.495, *p* < 0.001), LDH (r = −0.355, *p* < 0.001), CHO (r = −0.349, *p* < 0.001), Hcy (r = −0.293, *p* < 0.01), sperm TEAC (r = −0.295, *p* < 0.01) and CEA (r = −0.258, *p* < 0.05).

Sperm vitality showed significant inverse correlations with CK (r = −0.486, *p* < 0.001), LDH (r = −0.380, *p* < 0.001), ALB (r = −0.260, *p* < 0.05), IL-6 (r = −0.233, *p* < 0.05) and Hcy (r = −0.241, *p* < 0.05). Like normal sperm morphology, vitality showed a significant positive correlation with TPSA (r = 0.215, *p* < 0.05).

In Table 3, the detected values of ALB, CEA, CHO, CK, IL-6, LDH, TPSA, UA, Hcy, sperm TEAC and seminal TEAC were compared between individuals with sperm progressive motility below the 25th percentile (PMOT < 25 group; n = 28) and those with sperm progressive motility ≥ 25th percentile (PMOT ≥ 25 group; n = 64), between individuals with normal sperm morphology below the 25th percentile (MOR < 25 group; n = 32) and those with normal sperm morphology ≥ 25th percentile (MOR ≥ 25 group; n = 60), and between individuals with sperm vitality below the 25th percentile (VIT < 25 group; n = 29) and those with sperm vitality ≥ 25th percentile (VIT ≥ 25 group; n = 63). As expected, progressive sperm motility (53 vs. 32%, *p* < 0.001, Table 3), normal sperm morphology (10 vs. 5%, *p* < 0.001, Table 3) and sperm vitality (76 vs. 60%, *p* < 0.001, Table 3) were increased in PMOT ≥ 25 group, MOR ≥ 25 group, and VIT ≥ 25 group, respectively.

PMOT < 25 showed significantly higher ALB levels (0.72 vs. 0.51 g/dL, *p* < 0.001), CHO (23.96 vs. 13.35 mg/dL, *p* < 0.001), LDH (2140 vs. 1467 U/L, *p* < 0.001), Hcy (4.42 vs. 2.95 µmol/L, *p* < 0.001) and sperm TEAC (0.16 vs. 0.06 mM, *p* < 0.01, Table 3) compared with subjects with PMOT ≥ 25. Conversely, men with PMOT ≥ 25 showed significantly increased TPSA (16.60 vs. 13.70 ng/mL, *p* < 0.05) level, sperm concentration (46 vs. 35 ×10^6^/mL, *p* < 0.001, Table 3), normal sperm morphology (10 vs. 5%, *p* < 0.001, Table 3) and sperm vitality (75 vs. 71%, *p* < 0.05, Table 3).

Subjects with MOR < 25 exhibited significantly higher ALB levels (0.72 vs. 0.52 g/dL, *p* < 0.001), CHO (21.33 vs. 15.07 mg/dL, *p* < 0.01), CK (428 vs. 244 U/L, *p* < 0.05), LDH (2140 vs. 1467 U/L, *p* < 0.001), Hcy (3.98 vs. 2.95 µmol/L, *p* < 0.01) and sperm TEAC (0.16 vs. 0.06 mM, *p* < 0.001) compared with men with MOR ≥ 25. In contrast, men with MOR ≥ 25 showed significantly higher TPSA levels (16.27 vs. 13.90 ng/mL, *p* < 0.05), sperm concentration (47 vs. 36 ×10^6^/mL, *p* < 0.001, Table 3), progressive motility (53 vs. 35%, *p* < 0.001, Table 3), and sperm vitality (76 vs. 68%, *p* < 0.001, Table 3).

Subjects with VIT < 25 displayed significantly higher ALB levels (0.66 vs. 0.53 g/dL, *p* < 0.01), CK (591 vs. 219 U/L, *p* < 0.001, Table 3), LDH (2104 vs. 1541 U/L, *p* < 0.01), Hcy (3.73 vs. 2.95 µmol/L, *p* < 0.05) compared with subjects with VIT ≥ 25. Differently, men with VIT ≥ 25 showed significantly higher TPSA levels (16.66 vs. 14.90 ng/mL, *p* < 0.05), normal sperm morphology (9 vs. 6%, *p* < 0.001, Table 3). Across the three stratifications, no significant differences were observed for semen volume, CEA, CK, IL-6, UA and seminal TEAC in the PMOT < 25 versus PMOT ≥ 25 comparison; for semen volume, CEA, IL-6, UA and seminal TEAC in the MOR < 25 versus MOR ≥ 25 comparison; and for semen volume, sperm concentration, progressive motility, CEA, CHO, IL-6, UA and seminal TEAC in the VIT < 25 versus VIT ≥ 25 comparison.

**Figure 2:**
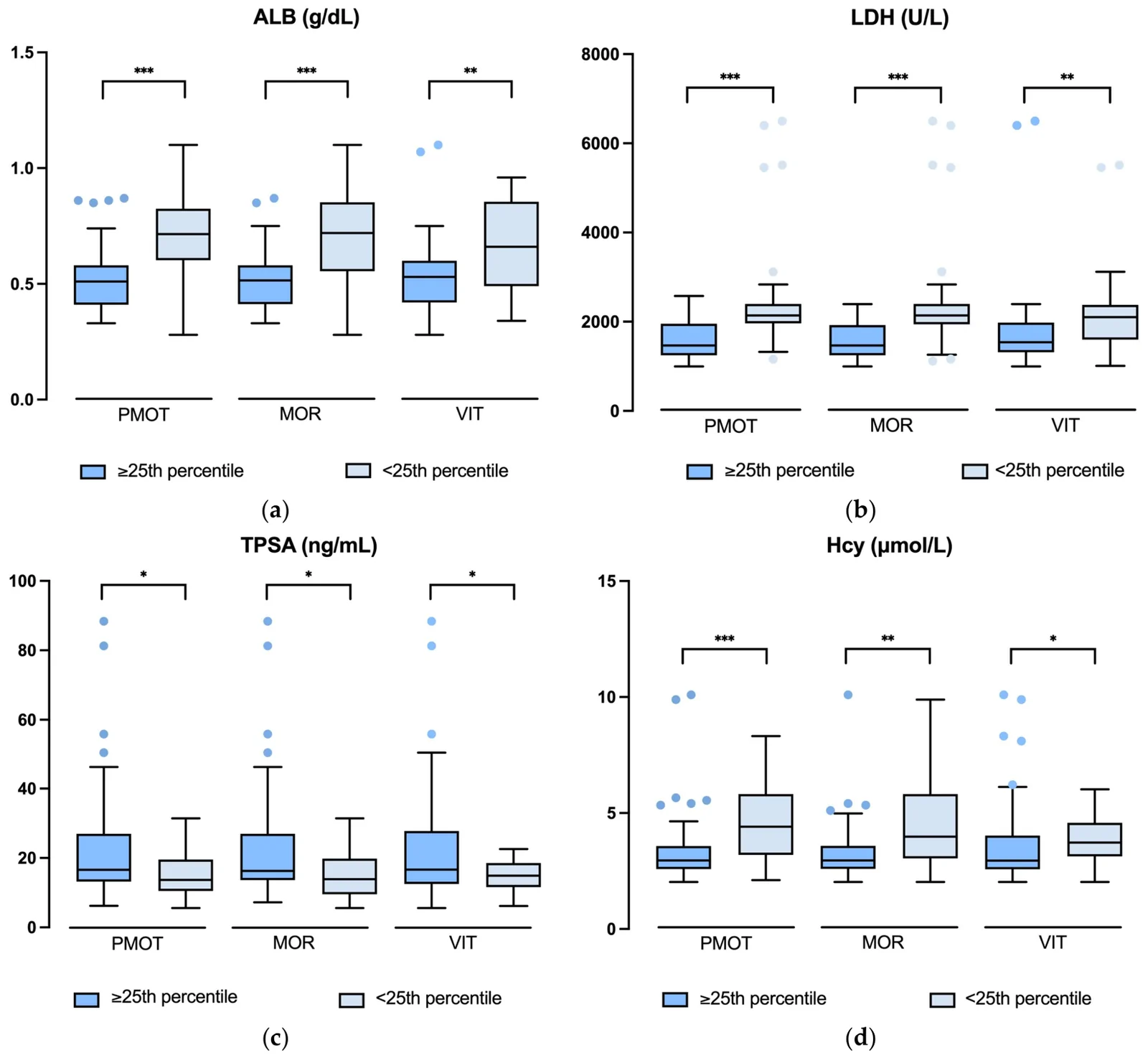
Box-and-whisker plots of seminal biomarkers that remained significantly different across all semen parameter stratifications. Seminal levels of (**a**) albumin (ALB, g/dL), (**b**) lactate dehydrogenase (LDH, U/L), (**c**) total prostate-specific antigen (TPSA, ng/mL) and (**d**) homocysteine (Hcy, µmol/L) are shown in subjects below the 25th percentile (<25th percentile) and at or above the 25th percentile (≥25th percentile) for progressive sperm motility (PMOT), normal sperm morphology (MOR) and sperm vitality (VIT). Median values, interquartile ranges and outliers are shown. Statistical significance between groups is indicated as follows: * *p* < 0.05, ** *p* < 0.01, *** *p* < 0.001.

Figure 2 showed the variables significantly different in all the three groups; in particular ALB, LDH, and Hcy levels were significantly increased in PMOT < 25, MOR < 25 and VIT < 25 groups and TPSA was significantly increased in PMOT ≥ 25, MOR ≥ 25 and VIT ≥ 25 groups.

As a second part of the study, the population was stratified to evaluate variations in seminal plasma biomolecule composition according to combined semen quality profiles defined by the three investigated sperm parameters. Specifically, subjects were classified into three groups: high-parameter group (H, all three parameters ≥ 25th percentile), intermediate-parameter group (I, two out of three parameters < 25th percentile) and low-parameter group (L, all three parameters < 25th percentile). Considering the relatively small size of the low-parameter group (L, n = 11), findings related to this subgroup should be interpreted with caution.

**Figure 3:**
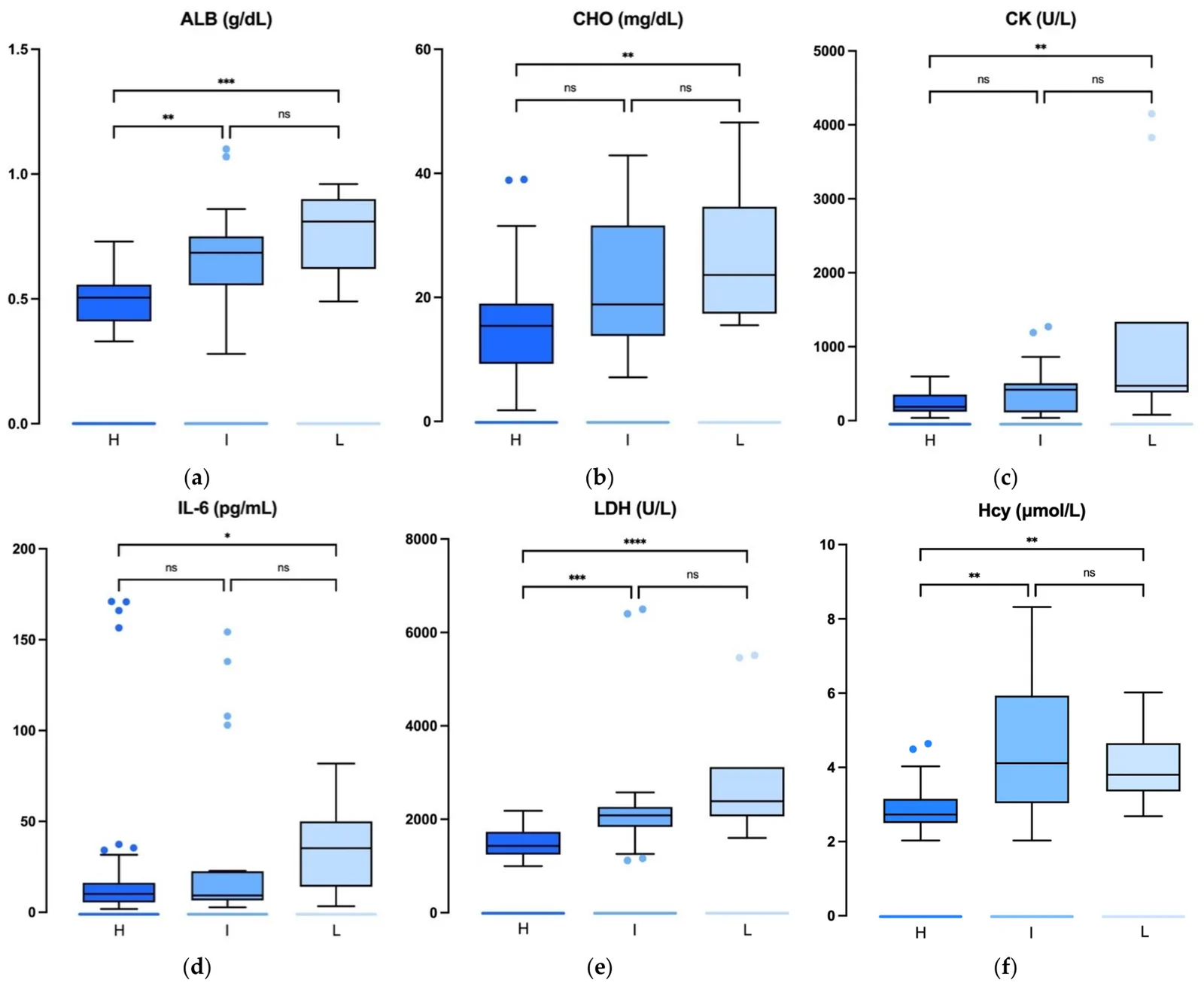

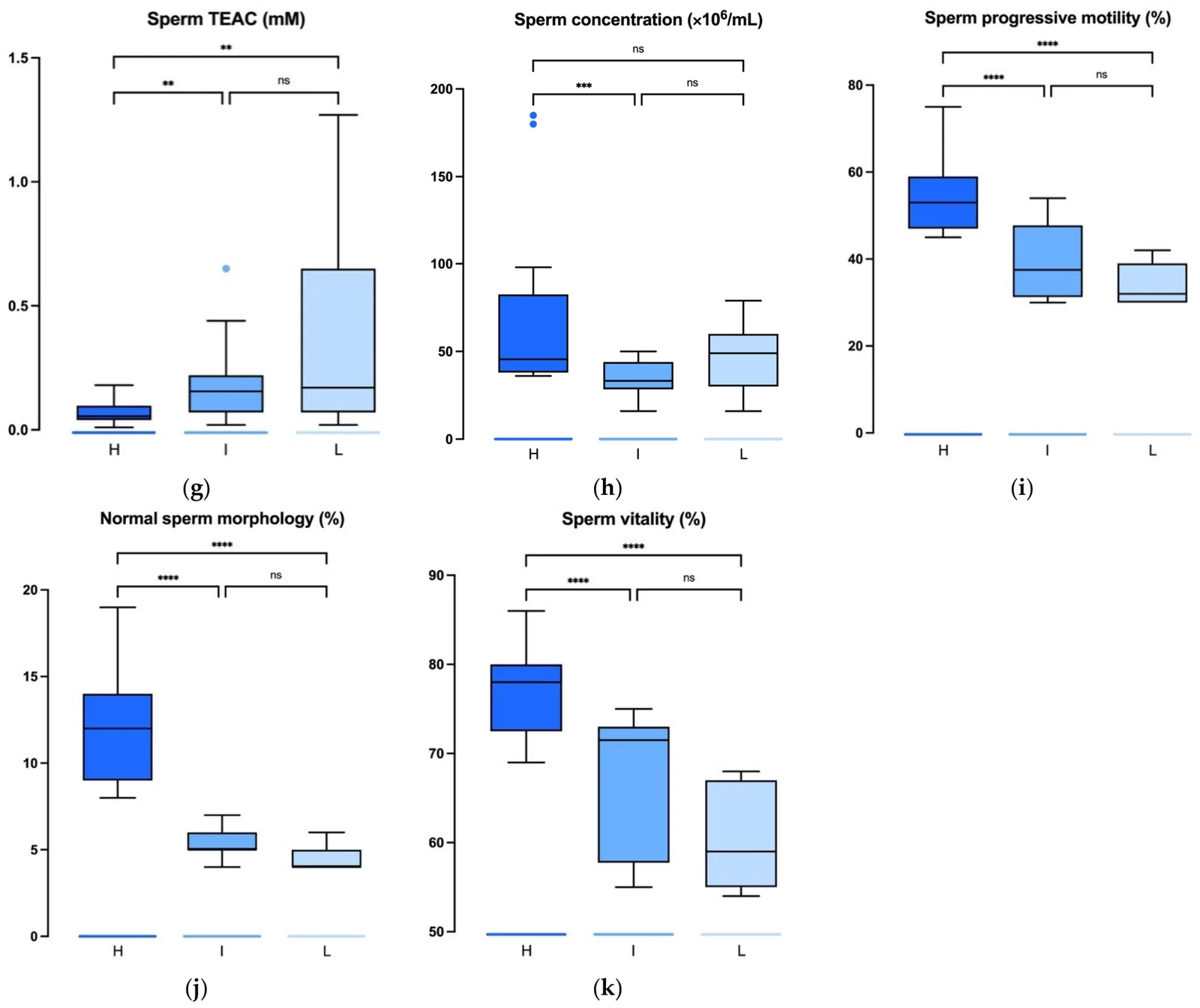
Box-and-whisker plots showing parameters with statistically significant differences among high-parameter, intermediate-parameter and low-parameter groups (respectively indicated as H, I, L), including (**a**) albumin (ALB, g/dL), (**b**) cholesterol (CHO, mg/dL), (**c**) creatine kinase (CK, U/L), (**d**) interleukin-6 (IL-6, pg/mL), (**e**) lactate dehydrogenase (LDH, U/L), (**f**) homocysteine (Hcy, µmol/L) and (**g**) sperm Trolox Equivalent Antioxidant Capacity (sperm TEAC, mM), (**h**) sperm concentration (×10^6^/mL), (**i**) sperm progressive motility (%), (**j**) normal sperm morphology (%), and (**k**) sperm vitality (%). Median values, interquartile ranges, and outliers are shown. Statistical significance between groups is indicated as follows: ns non-significant, * *p* < 0.05, ** *p* < 0.01, *** *p* < 0.001, **** *p* < 0.0001.

As reported in Table 4, subjects in the L group showed significantly higher ALB levels (0.81 vs. 0.51 g/dL, *p* < 0.001, Figure 3a), CHO (23.65 vs. 15.40 mg/dL, *p* < 0.01, Figure 3b), CK (472 vs. 187 U/L, *p* < 0.01, Figure 3c), IL-6 (35.30 vs. 10.15 pg/mL, *p* < 0.05, Figure 3d), LDH (2386 vs. 1431 U/L, *p* < 0.001, Figure 3e), Hcy (3.80 vs. 2.73 µmol/L, *p* < 0.01, Figure 3f) and sperm TEAC (0.17 vs. 0.06 mM, *p* < 0.01, Figure 3g) compared with subjects in the H group. Moreover, the I group also showed significantly higher ALB (0.68 vs. 0.51 g/dL, *p* < 0.01, Figure 3a), LDH (2083 vs. 1431 U/L, *p* < 0.001, Figure 3e), Hcy (4.12 vs. 2.73 µmol/L, *p* < 0.01, Figure 3f) and sperm TEAC (0.16 vs. 0.06 mM, *p* < 0.01, Figure 3g) compared with the H group. No significant differences were observed for CEA, TPSA, UA and seminal TEAC.

## 4. Discussion

Seminal components, nutrients and molecular signals act as dynamic regulators of sperm function and reproductive competence [38,39]; OS and inflammatory processes are recognized as key determinants involved in altering delicate biochemical homeostasis [39]. In this context, the present study investigated the seminal biochemical profile of men with semen parameters above the 5th percentile according to WHO criteria [36], highlighting significant associations between several seminal plasma biomarkers, antioxidant capacity, and sperm parameters. Overall, the findings suggest that most seminal plasma biomolecules follow a specific trend in relation to sperm parameters, indicating a close relationship between the seminal biochemical environment and sperm function within a population not classified as infertile according to conventional reference limits [36].

To investigate the relationships between the biochemical markers measured in seminal plasma, sperm TEAC, and conventional semen parameters, three analytical approaches were adopted: (1) correlation analyses among all variables considering the entire study population; (2) stratification of samples using normal progressive sperm motility, normal sperm morphology, and vitality as discriminating parameters, classified above or below the 25th percentile; and (3) classification into three groups: H group, in which all three sperm parameters were above the 25th percentile; I group, in which two out of three parameters ranged between the 5th and 25th percentiles; and L group, in which all three parameters fell within the 5th–25th percentile range.

Overall, ALB, CHO, LDH, Hcy and sperm TEAC increase with a decrease in sperm parameters. These molecules, which displayed strong mutual correlations, were also associated with variations in sperm parameters within the physiological range, as the 5th percentile values established by the WHO guidelines [36] represent the clinical thresholds used to distinguish suboptimal semen quality from values considered normal. After stratification, the L group showed significantly increased levels of ALB, CHO, CK, IL-6, LDH, Hcy, and sperm TEAC compared to H group. The levels of some of these molecules also differed between H and I group, except for CHO, CK, and IL-6, further demonstrating the existence of a correlation and a progressive gradient in their levels in relation to sperm parameters.

The increase of ALB and CHO may alter membrane dynamics or mild oxidative conditions probably affecting sperm function. In particular, ALB was negatively correlated with normal sperm morphology, motility and vitality, indicating possible mild alteration of the reproductive system at different levels [6,40]. ALB is physiologically present in seminal plasma and contributes to osmotic balance and molecule transport; however, increased seminal ALB concentrations may reflect altered permeability of the blood–testis or accessory gland barriers [41]. Alternatively, given albumin’s role as a potent extracellular antioxidant and carrier molecule, its elevated presence might reflect a compensatory, protective mechanism aimed at safeguarding spermatozoa, rather than a definitive sign of barrier disruption or accessory gland alteration [42].

CHO plays a critical role in sperm membrane structure and capacitation processes. Excessive CHO retention in sperm membranes may reduce membrane fluidity, impair capacitation, and consequently compromise sperm motility and fertilizing ability [17].

Among the investigated biomarkers, LDH, a cytoplasmic oxidoreductase, is one of the strongest indicators associated with reduced sperm quality as observed also by Feng and colleagues [43]. In the male reproductive system, the LDH-C isoform is specifically expressed in germ cells and spermatozoa and is essential for sperm energy metabolism [19,20]. In humans, higher LDH and LDH-C activity is observed in oligozoospermic patients compared to normozoospermic individuals [44]; some recent studies suggest that the detection of LDH-C in seminal plasma may predict successful retrieval of viable testicular spermatozoa [5,27,45].

In our dataset, Hcy emerged as an important component associated with several other seminal molecules. Interestingly, the Hcy concentrations detected in the seminal plasma of the studied normozoospermic population were slightly increased, although comparable to those previously reported in other studies [46,47], suggesting that a moderate increase of this metabolite may occur even in individuals with semen parameters within the normal range according to WHO criteria [36]. Clinically, seminal Hcy has been negatively associated with embryo quality in IVF/ICSI procedures [48], and infertile men show significantly higher seminal plasma Hcy compared with fertile controls, with these levels inversely correlated with sperm concentration and motility [49]. Some authors have suggested that elevated Hcy levels may increase ROS production due to the high reactivity of its thiol group and reduce nitric oxide bioavailability, thereby impairing sperm motility [50]. Importantly, Hcy metabolism is tightly regulated by folate- and vitamin B12-dependent pathways: deficiencies or imbalances in these micronutrients may lead to Hcy accumulation, contributing to the development of a pro-oxidative intracellular environment [46,47,51]. Taken together, these findings seem to suggest that even small alterations in Hcy metabolism may contribute to mild oxidative imbalance within the seminal microenvironment.

Inflammatory mediators further contribute to seminal biochemical alterations: IL-6 has been associated with oxidative damage in spermatozoa and impaired semen quality [22]. Experimental evidence also suggests that IL-6 may interfere with sperm mitochondrial activity via inducible nitric oxide synthase pathways [52], potentially affecting sperm motility and vitality. In this study, the levels of IL-6 increased in semen samples with the L group; moreover, the negative correlation between IL-6 and sperm vitality and the positive correlations observed between IL-6 vs. both CK and LDH support an association among inflammation, energy metabolism and cellular damage in the seminal microenvironment present also in normozoospermia.

Within this biochemical network, UA appears to play a relevant and multifaceted role. As one of the most abundant non-enzymatic antioxidants in seminal plasma, it contributes significantly to the ROS neutralization through free radical scavenging and transition metal chelation [28,29,31,32,37,53]. In our research, the median UA concentration across the entire cohort was similar to that reported in seminal plasma of normozoospermic men by Janiszewska and colleagues [32]. UA showed positive correlations with TPSA, seminal TEAC, Hcy, IL-6 and CEA, supporting its involvement in a tightly interconnected network linking OS, inflammation and accessory gland activity. The association with Hcy is particularly noteworthy, as elevated Hcy levels are known to contribute to a pro-oxidative environment, suggesting that increased UA levels may reflect a compensatory response to metabolically driven OS. Similarly, the positive association with IL-6 and CEA indicates that UA levels may rise in parallel with low-grade inflammation and/or mild OS. Notably, the positive relationship between UA and seminal TEAC confirms its contribution to total antioxidant capacity. However, this finding should be interpreted with caution, as UA may exert both antioxidant and pro-oxidant effects depending on its concentration and the surrounding biochemical context [31,32].

When considered together, the observed correlations among seminal biomarkers highlight the existence of an interconnected biochemical network rather than independent alterations of individual molecules. The relationships involving ALB, CHO, CK, LDH, Hcy, IL-6, UA, TPSA, CEA and sperm TEAC suggest the simultaneous involvement of multiple biological processes, including membrane remodeling, energy metabolism, inflammatory signaling, oxidative balance and accessory gland activity. In particular, the association of ALB, CHO, CK and LDH with Hcy, IL-6 and sperm TEAC suggests that alterations in seminal biochemical homeostasis may arise from the interaction between metabolic stress, inflammation and redox regulation. Conversely, the relationships observed with TPSA, CEA and UA indicate the contribution of accessory gland secretory activity to this complex environment. Thus, the observed correlation pattern supports the interpretation that sperm quality within the normozoospermic range is influenced by a coordinated biochemical milieu, in which moderate variations in multiple seminal components may collectively contribute to differences in sperm functional efficiency.

Stratifying the study population according to the quality of individual sperm parameters, namely motility, normal morphology, and vitality, ALB, Hcy, LDH concentrations were increased in the groups with sperm progressive motility, normal morphology and vitality < 25th percentile. CK levels were increased in the groups with reduced normal sperm morphology and vitality as well as in I and L groups, confirming its role as a marker of cellular damage. CK is a key enzyme in maintaining adequate ATP availability for sperm motility [18]. Previous studies have reported increased CK activity in semen from patients with infertility-related conditions such as varicocele or idiopathic infertility, often in association with OS and impaired sperm parameters [54] and from patients with sperm vitality ≤ 5th percentile [6]. Additionally, CHO levels were higher in groups characterized by lower motility and viability, indicating alterations of the sperm membrane.

An intriguing finding concerns antioxidant capacity. Sperm TEAC is a more significant marker than seminal TEAC, as recently demonstrated by Signorini and colleagues [35]. Mature spermatozoa have limited intrinsic antioxidant defenses due to the cytoplasmic loss that occurs during spermiogenesis. Consequently, they rely mainly on the antioxidant capacity of seminal plasma, although residual intracellular antioxidant systems remain essential for the maintenance of proper sperm function [33]. The seminal TEAC levels observed in our study are similar to those previously reported in studies comparing fertile and infertile men [35,55,56,57]. Recently, total antioxidant activity has also been used to evaluate molecular alterations in semen due to environmental factors [58].

Sperm TEAC was higher in subjects with lower sperm quality and negatively correlated with sperm motility and normal morphology. At first glance, this observation may appear counterintuitive, since antioxidant capacity is generally considered protective. However, increased sperm TEAC may represent a compensatory response to mild OS rather than a marker of improved redox balance. In conditions characterized by moderate oxidative damage, spermatozoa may upregulate antioxidant defenses in an attempt to counteract ROS generation. The concomitant increase in Hcy, LDH, and inflammatory markers supports this interpretation. Similar findings have been reported by Shcherbitskaia and colleagues [29], who observed an association between elevated cellular TEAC levels and an increased DNA fragmentation index.

Notably, TPSA was the only parameter, among those analyzed in this study, associated with the improvement of sperm parameters. The positive role of TPSA appears to be confirmed by other studies [9,26]. TPSA is a serine protease produced by the prostate that promotes the liquefaction of seminal coagulum proteins after ejaculation, thereby facilitating sperm release and motility within the female reproductive tract. In our dataset, TPSA showed positive correlations with UA, and higher TPSA concentrations were consistently observed in groups with better motility, morphology, and vitality, while inverse correlations were found with ALB, CHO, CK, and LDH. Since TPSA contributes to semen liquefaction and reflects prostate secretory function [59], these findings may indicate that preserved prostate activity supports an optimal seminal environment and sperm function. Reduced TPSA levels could therefore reflect a suboptimal accessory gland function, with the observed correlation indicating that poorer accessory gland function is associated with lower semen quality.

The results of this study suggest that the WHO lower reference limits mark the lower end of a broad functional range, rather than a cut-off between normal and abnormal reproductive function: within this range, individuals may exhibit various degrees of biochemical efficiency and overall sperm function, which are closely correlated, demonstrating a strong relationship between the two major components of seminal fluid, namely spermatozoa and seminal plasma. Early molecular signs of metabolic or OS may be present in individuals at the lower end of the range, before any measurable changes in sperm parameters are observed.

This interpretation suggests several directions for future research. First, longitudinal studies are needed to define whether individuals with this biochemical profile are more likely to experience declines in semen quality, clarifying if these molecular differences are temporary variations or early signs of reproductive risk. Second, combining these biochemical markers with functional sperm tests, such as mitochondrial activity, lipid peroxidation, sperm chromatin integrity and proteomic or metabolomic profiling, could show whether these molecular changes affect sperm function in ways not detected by routine semen analysis. Third, systemic factors that influence OS and metabolism, such as diet, metabolic syndrome, physical activity and micronutrient status, should be studied in relation to these biochemical patterns. Finally, evaluating seminal biochemical markers alongside standard semen parameters could help develop more sensitive tools for assessing male reproductive function. Some limitations of this research should be acknowledged. Since the study has a cross-sectional design, data were collected at a single time point without following subjects over time. Thus, it is possible to observe associations or correlations between biomarkers and sperm dysfunction, but it is not possible to demonstrate a cause-and-effect relationship. Moreover, OS was assessed indirectly through TEAC rather than through direct quantification of ROS or oxidative damage products. Finally, the study population included only men with semen parameters above WHO reference limits; therefore, the findings cannot be directly generalized to infertile populations with severe semen abnormalities.

In addition, the exploratory nature of the study and the relatively high number of investigated variables compared with the sample size limited the application of multivariable analyses. These findings provide a basis for future investigations aimed at further exploring the independent role of individual biomarkers.

## 5. Conclusions

**Figure 4:**
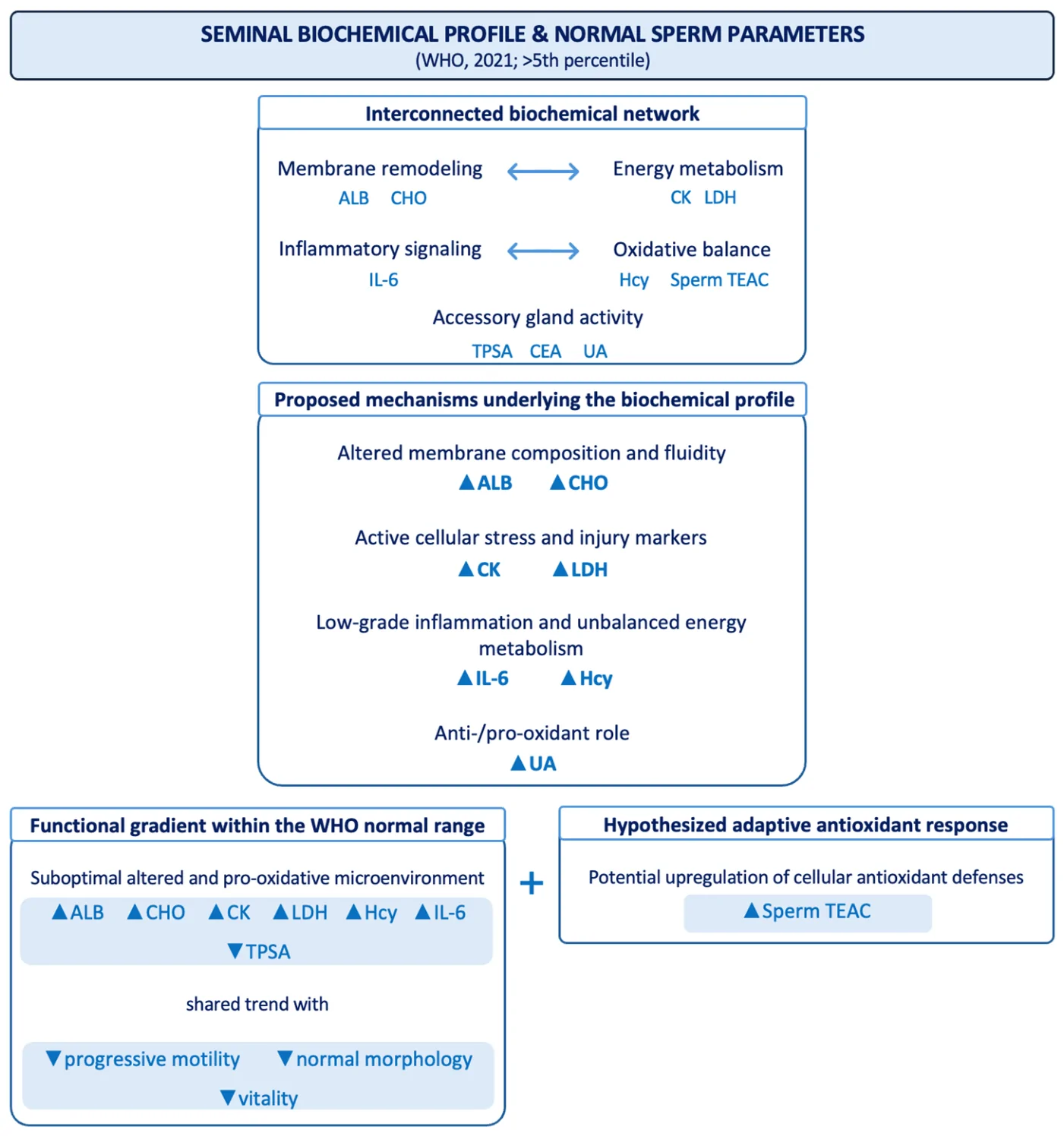
Schematic summary of the seminal biochemical profile associated with normal sperm parameters. The diagram summarizes the main findings of the study and their proposed biological interpretation. The biochemical profile observed in seminal plasma of normozoospermic men (>5th percentile, [36]) suggests interconnected changes involving membrane remodeling, energy metabolism, inflammatory signaling, oxidative balance and accessory gland activity. Increased ALB and CHO may reflect altered membrane composition and fluidity, whereas elevated CK and LDH are consistent with cellular stress and tissue injury. Increased IL-6 and Hcy suggest a low-grade inflammatory and pro-oxidant environment, while UA may contribute to redox regulation. Collectively, these changes define a functional biochemical gradient within the WHO normal range, associated with lower progressive motility, normal morphology, vitality and TPSA levels. The concomitant increase in sperm TEAC may reflect a hypothesized adaptive antioxidant response.

In conclusion, these results suggest the coexistence of multiple mild sperm impairments with a progressive alteration of the seminal biochemical milieu. Normal sperm morphology and progressive sperm motility appear to be the most sensitive indicators of seminal biochemical variations, strongly associated with markers of cellular damage, OS and inflammation. The observed increase in sperm antioxidant capacity (sperm TEAC) could reflect a compensatory response rather than effective protection. Conversely, higher TPSA concentrations were associated with a more favorable seminal biochemical profile. The overall interpretation of the observed biochemical profile is summarized in Figure 4.

## Figures and Tables

**Table 1:** Medians and interquartile ranges (IQR: 25th–75th percentile) of seminal albumin (ALB, g/dL), carcinoembryonic antigen (CEA, ng/mL), cholesterol (CHO, mg/dL), creatine kinase (CK, U/L), interleukin-6 (IL-6, pg/mL), lactate dehydrogenase (LDH, U/L), total prostate-specific antigen (TPSA, ng/mL), uric acid (UA, mg/dL), homocysteine (Hcy, µmol/L), semen volume (mL), sperm concentration (×10^6^/mL), sperm progressive motility (%), normal sperm morphology (%), sperm vitality (%), seminal and sperm Trolox Equivalent Antioxidant Capacity (seminal, sperm TEAC, mM) are reported.

	Median	IQR
ALB (g/dL)	0.55	0.44–0.70
CEA (ng/mL)	20.58	9.66–52.71
CHO (mg/dL)	16.30	9.74–25.60
CK (U/L)	320	134–482
IL-6 (pg/mL)	12.15	6.59–30.71
LDH (U/L)	1798	1327–2132
TPSA (ng/mL)	15.70	12.14–23.09
UA (mg/dL)	5.1	4.2–6.5
Hcy (μmol/L)	3.17	2.67–4.44
Volume (mL)	3.5	2.5–4.0
Sperm concentration (×10^6^/mL)	44	36–69
Sperm progressive motility (%)	49	40–58
Normal sperm morphology (%)	9	6–11
Sperm vitality (%)	72	66–78
Sperm TEAC (mM)	0.08	0.04–0.15
Seminal TEAC (mM)	2.05	1.83–2.10

**Table 2:** Spearman correlation coefficients among seminal albumin (ALB, g/dL), carcinoembryonic antigen (CEA, ng/mL), cholesterol (CHO, mg/dL), creatine kinase (CK, U/L), interleukin-6 (IL-6, pg/mL), lactate dehydrogenase (LDH, U/L), total prostate-specific antigen (TPSA, ng/mL), uric acid (UA, mg/dL), homocysteine (Hcy, µmol/L), semen volume (mL), sperm concentration (×10^6^/mL), sperm progressive motility (%), normal sperm morphology (%), sperm vitality (%) and seminal and sperm Trolox Equivalent Antioxidant Capacity (seminal, sperm TEAC, mM). Significant correlations are reported: * *p* < 0.05, ** *p* < 0.01, *** *p* < 0.001.

	ALB	CEA	CHO	CK	IL-6	LDH	TPSA	UA	Hcy	Semen Volume	Sperm Concentration	Sperm Progressive Motility	Normal Sperm morphology	Sperm Vitality	Sperm TEAC	Seminal TEAC
ALB (g/dL)	1															
CEA (ng/mL)	−0.086	1														
CHO (mg/dL)	0.377 ***	−0.118	1													
CK (U/L)	0.163	−0.188	−0.016	1												
IL-6 (pg/mL)	0.160	0.009	0.027	0.305 **	1											
LDH (U/L)	0.577 ***	−0.061	0.582 ***	0.157	0.228 *	1										
TPSA (ng/mL)	−0.425 ***	0.245 *	−0.530 ***	−0.311 **	0.025	−0.457 ***	1									
UA (mg/dL)	−0.083	0.233 *	−0.346 ***	−0.071	0.221 *	−0.107	0.338 ***	1								
Hcy (μmol/L)	0.426 ***	0.209 *	0.042	0.026	0.113	0.254 *	0.006	0.320 **	1							
Semen volume (mL)	0.017	0.161	0.033	−0.160	−0.087	−0.191	0.184	0.128	−0.177	1						
Sperm concentration (×10^6^/mL)	−0.127	−0.127	−0.038	−0.201	−0.166	0.066	0.103	−0.039	−0.159	−0.192	1					
Sperm progressive motility (%)	−0.495 ***	−0.258 *	−0.349 ***	−0.029	−0.078	−0.355 ***	0.173	0.128	−0.293 **	−0.095	0.475 ***	1				
Normal sperm morphology (%)	−0.515 ***	−0.207 *	−0.386 ***	−0.294**	−0.005	−0.514 ***	0.258 *	0.081	−0.381 ***	0.046	0.259 *	0.590 ***	1			
Sperm vitality (%)	−0.260 *	0.102	0.014	−0.486 ***	−0.233 *	−0.380 ***	0.215 *	−0.106	−0.241 *	0.199	0.106	0.128	0.449 ***	1		
Sperm TEAC (mM)	0.174	0.191	0.223 *	0.248 *	0.095	0.105	−0.021	0.024	0.146	0.182	−0.213 *	−0.295 **	−0.438 ***	−0.168	1	
Seminal TEAC (mM)	−0.109	0.026	−0.123	−0.005	−0.213 *	−0.086	0.123	0.221 *	−0.050	0.034	0.185	0.191	−0.054	−0.052	0.170	1

**Table 3:** Medians and interquartile ranges (IQR: 25th–75th percentile) of seminal albumin (ALB, g/dL), carcinoembryonic antigen (CEA, ng/mL), cholesterol (CHO, mg/dL), creatine kinase (CK, U/L), interleukin-6 (IL-6, pg/mL), lactate dehydrogenase (LDH, U/L), total prostate-specific antigen (TPSA, ng/mL), uric acid (UA, mg/dL), homocysteine (Hcy, µmol/L), semen volume (mL), sperm concentration (×10^6^/mL), sperm progressive motility (%), normal sperm morphology (%), sperm vitality (%), seminal and sperm Trolox Equivalent Antioxidant Capacity (seminal, sperm TEAC, mM) in men with sperm progressive motility < 25th percentile (PMOT < 25, n = 28) versus men with sperm progressive motility ≥ 25th percentile (PMOT ≥ 25, n = 64), men with normal sperm morphology < 25th (MOR < 25, n = 32) versus men with sperm normal morphology ≥ 25th (MOR ≥ 25, n = 60) and men with sperm vitality < 25th (VIT < 25, n = 29) versus men with sperm vitality ≥ 25th (VIT ≥ 25, n = 63). Statistics are also reported: * *p* < 0.05, ** *p* < 0.01, *** *p* < 0.001.

	Sperm Progressive Motility		Normal Sperm Morphology		Sperm Vitality	
Variable	PMOT < 25	PMOT ≥ 25	MOR < 25	MOR ≥ 25	VIT < 25	VIT ≥ 25
ALB (g/dL)	0.72 *** (0.60–0.82)	0.51 (0.41–0.58)	0.72 *** (0.56–0.84)	0.52 (0.42–0.58)	0.66 ** (0.49–0.85)	0.53 (0.43–0.60)
CEA (ng/mL)	43.58 (9.03–86.39)	17.83 (9.88–24.80)	22.32 (9.03–65.61)	20.08 (9.98–34.62)	18.79 (9.21–24.80)	21.10 (11.04–55.00)
CHO (mg/dL)	23.96 *** (17.30–27.98)	13.35 (8.85–19.20)	21.33 ** (15.77–29.53)	15.07 (8.96–20.57)	17.40 (7.20–27.29)	16.26 (11.25–22.12)
CK (U/L)	395 (212–474)	270 (133–487)	428 * (263–575)	244 (125–381)	591 *** (382–1177)	219 (122–361)
IL-6 (pg/mL)	17.96 (9.20–37.59)	11.48 (5.47–26.04)	13.90 (8.32–41.07)	11.55 (5.46–28.55)	18.70 (6.59–35.30)	10.80 (6.87–22.90)
LDH (U/L)	2140 *** (1974–2396)	1467 (1256–1928)	2140 *** (1952–2391)	1467 (1254–1924)	2104 ** (1601–2374)	1541 (1320–1975)
TPSA (ng/mL)	13.70 * (10.93–18.85)	16.60 (13.60–26.00)	13.90 * (10.05–19.60)	16.27 (13.63–26.33)	14.90 * (11.67–18.26)	16.66 (12.80–27.60)
UA (mg/dL)	4.9 (3.8–6.2)	5.2 (4.4–6.9)	5.0 (4.0–6.5)	5.2 (4.3–6.8)	5.9 (4.3–6.8)	4.9 (4.1–6.5)
Hcy (μmol/L)	4.42 *** (3.21–5.76)	2.95 (2.59–3.57)	3.98 ** (3.06–5.76)	2.95 (2.59–3.58)	3.73 * (3.17–4.51)	2.95 (2.59–3.98)
Volume (mL)	3.6 (3.00–4.00)	3.50 (2.5–4.0)	3.5 (2.6–4.0)	3.5 (2.5–4.0)	3.5 (2.5–3.9)	3.6 (2.5–4.0)
Sperm concentration (×10^6^/mL)	35 *** (31–49)	46 (38–84)	36 *** (30–49)	47 (38–84)	45 (31–76)	42 (36–68)
Sperm progressive motility (%)	32 *** (30–38)	53 (48–60)	35 *** (31–41)	53 (47–60)	50 (37–58)	48 (41–58)
Normal sperm morphology (%)	5 *** (4–6)	10 (9–12)	5 *** (4–6)	10 (9–13)	6 *** (4–9)	9 (8–13)
Sperm vitality (%)	71 * (60–73)	75 (67–80)	68 *** (57–72)	76 (70–80)	60 *** (55–65)	76 (72–80)
Sperm TEAC (mM)	0.16 ** (0.06–0.29)	0.06 (0.04–0.11)	0.16 *** (0.07–0.23)	0.06 (0.03–0.10)	0.12 (0.04– 0.18)	0.06 (0.04–0.13)
Seminal TEAC (mM)	2.02 (1.73–2.10)	2.05 (1.84–2.10)	2.06 (1.89–2.11)	2.05 (1.83–2.10)	2.09 (2.05–2.13)	2.05 (1.83–2.10)

**Table 4:** Medians and interquartile ranges (IQR: 25th–75th percentile) of seminal albumin (ALB, g/dL), carcinoembryonic antigen (CEA, ng/mL), cholesterol (CHO, mg/dL), creatine kinase (CK, U/L), interleukin-6 (IL-6, pg/mL), lactate dehydrogenase (LDH, U/L), total prostate-specific antigen (TPSA, ng/mL), uric acid (UA, mg/dL), homocysteine (Hcy, µmol/L), semen volume (mL), sperm concentration (×10^6^/mL), sperm progressive motility (%), normal sperm morphology (%), sperm vitality (%), seminal and sperm Trolox Equivalent Antioxidant Capacity (sperm TEAC, mM) in high-parameter group (H, n = 44) including men with sperm progressive motility, normal morphology and vitality ≥ 25th percentile (respectively ≥45%, ≥8%, and ≥69%), intermediate-parameter group (I, n = 20) including men with two out of the three among sperm progressive motility, normal morphology and vitality < 25th percentile (respectively <45%, <8%, and <69%), and low-parameter group (L, n = 11) including men with sperm progressive motility, normal morphology and vitality between the 5th and the 25th percentiles (respectively 30–44%, 4–7%, and 54–68%). Statistics are also reported: ns non-significant, * *p* < 0.05, ** *p* < 0.01, *** *p* < 0.001.

Variable	High Parameters (H)	Intermediate Parameters (I)	Low Parameters (L)	Statistics
ALB (g/dL)	0.51 (0.41–0.55)	0.68 (0.56–0.75)	0.81 (0.66–0.89)	H vs. I ** H vs. L ***
CEA (ng/mL)	18.42 (12.28–27.33)	18.25 (7.59–75.34)	23.00 (13.12–61.78)	ns
CHO (mg/dL)	15.40 (9.43–18.60)	18.83 (14.11–29.53)	23.65 (17.60–29.85)	H vs. L **
CK (U/L)	187 (122–351)	419 (166–488)	472 (395–1257)	H vs. L **
IL-6 (pg/mL)	10.15 (5.62–16.08)	9.24 (6.63–22.05)	35.30 (22.31–46.75)	H vs. L *
LDH (U/L)	1431 (1246–1671)	2083 (1902–2232)	2386 (2139–2976)	H vs. I *** H vs. L ***
TPSA (ng/mL)	18.50 (12.95–28.00)	14.43 (9.11–19.08)	12.74 (10.70–19.08)	ns
UA (mg/dL)	4.8 (4.1–6.0)	5.2 (4.2–6.5)	4.2 (3.8–6.1)	ns
Hcy (μmol/L)	2.73 (2.50–3.12)	4.12 (3.03–5.88)	3.80 (3.42–4.42)	H vs. I ** H vs. L **
Volume (mL)	3.5 (2.5–4.4)	3.6 (2.5–4.1)	3.0 (2.9–3.7)	ns
Sperm concentration (×10^6^/mL)	46 (38–79)	33 (29–44)	49 (31–59)	H vs. I ***
Progressive motility (%)	53 (47–59)	38 (31–47)	32 (30–38)	H vs. I *** H vs. L ***
Normal sperm morphology (%)	12 (9–14)	5 (5–6)	4 (4–5)	H vs. I *** H vs. L ***
Sperm vitality (%)	78 (73–80)	72 (59–73)	59 (55–63)	H vs. I *** H vs. L ***
Sperm TEAC (mM)	0.06 (0.04–0.09)	0.16 (0.09–0.22)	0.17 (0.07–0.64)	H vs. I ** H vs. L **
Seminal TEAC (mM)	2.05 (1.83–2.10)	2.07 (1.94–2.12)	2.05 (1.59–2.10)	ns

## Data Availability

The data presented in this study is available on request from the corresponding author. The data is not publicly available due to the privacy of the patients.

## Abbreviations

The following abbreviations are used in this manuscript:

WHO	World Health Organization
OS	Oxidative Stress
TEAC	Trolox Equivalent Antioxidant Capacity
ALB	Albumin
CHO	Cholesterol
CK	Creatine Kinase
LDH	Lactate Dehydrogenase
IL-6	Interleukin-6
Hcy	Homocysteine
CEA	Carcinoembryonic Antigen
ROS	Reactive Oxygen Species
TAC	Total Antioxidant Capacity
